# Supplementary material for: Comparative Genomic Analysis Reveals the Emergence of ST-231 and ST-395 Klebsiella pneumoniae Strains Associated with the High Transmissibility of blaKPC Plasmids
Source: Microorganisms. 2023 Sep 27;11(10):2411. doi: 10.3390/microorganisms11102411 (PMC10608898; doi:10.3390/microorganisms11102411)
Supplement: Supplementary file 1 [file microorganisms-11-02411-s001.zip › microorganisms-2618769-supplementary.pdf]

Supplementary figures

(A)

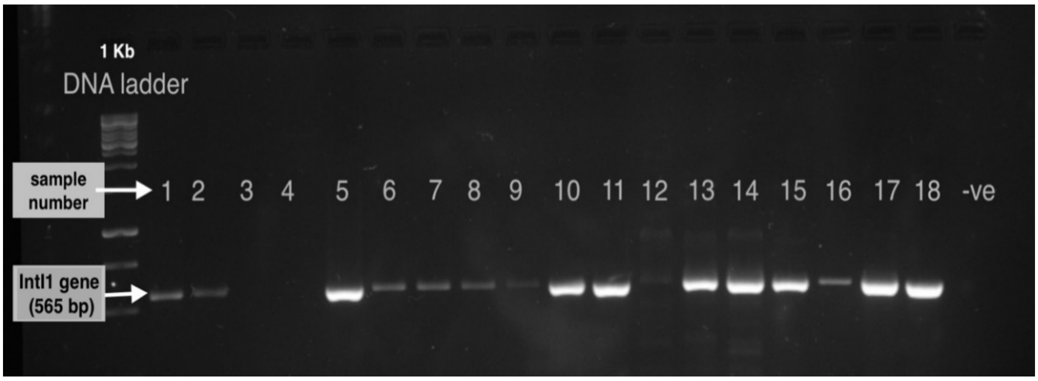

(B)

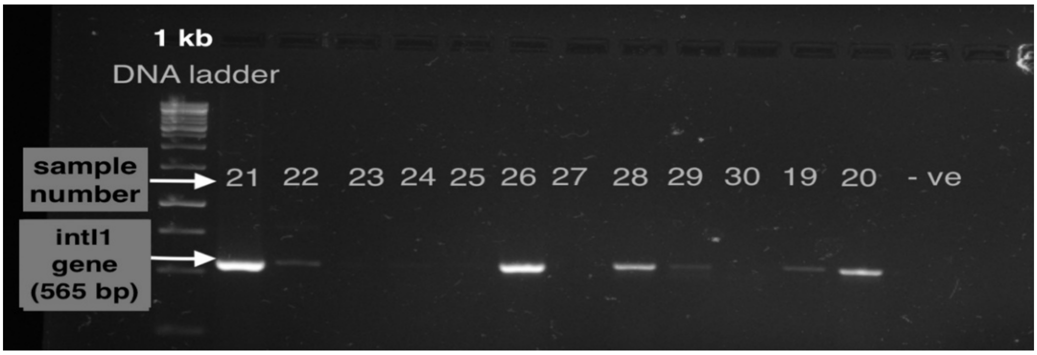

(C)

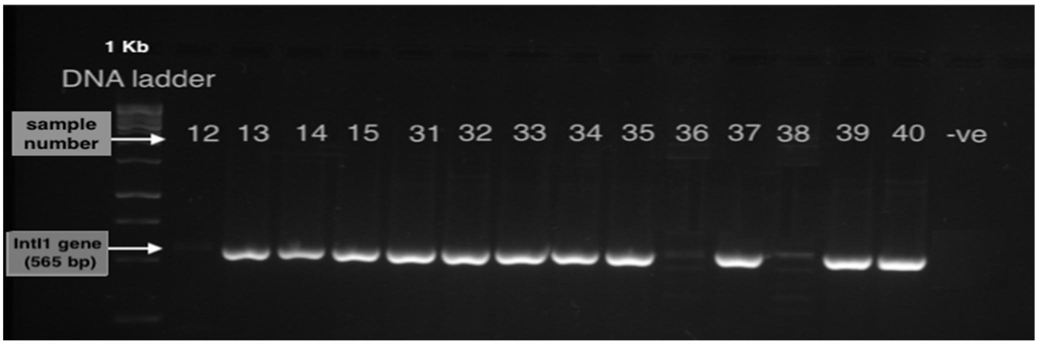

(D)

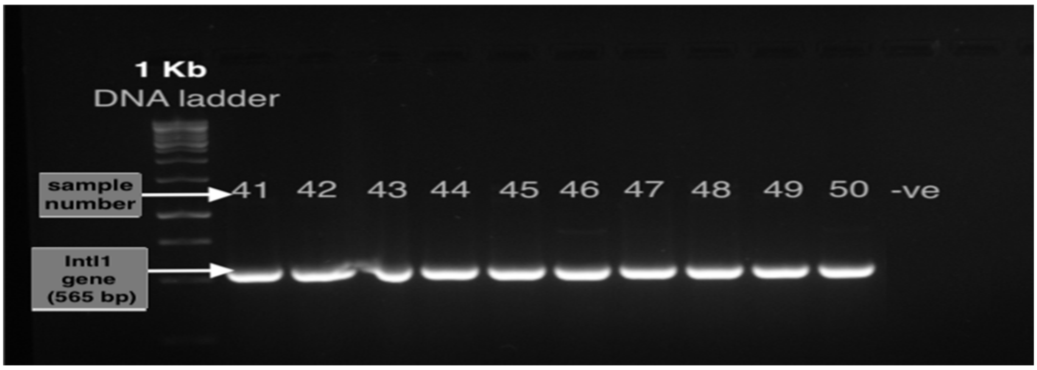

Supplementary Figure S1 Agarose gel electrophoresis of class 1 integrons (IntI1 gene).
(A, B, C, D) lane (1) 1 Kb DNA ladder, lane (2-19) *K. pneumoniae* isolates in **A**, (19-30) in **B**, (31-40) in **C** isolates 12, 13, 14 and 15 are repeated, and (41-50) in **D**. (-ve) negative control for integron gene.

(A)

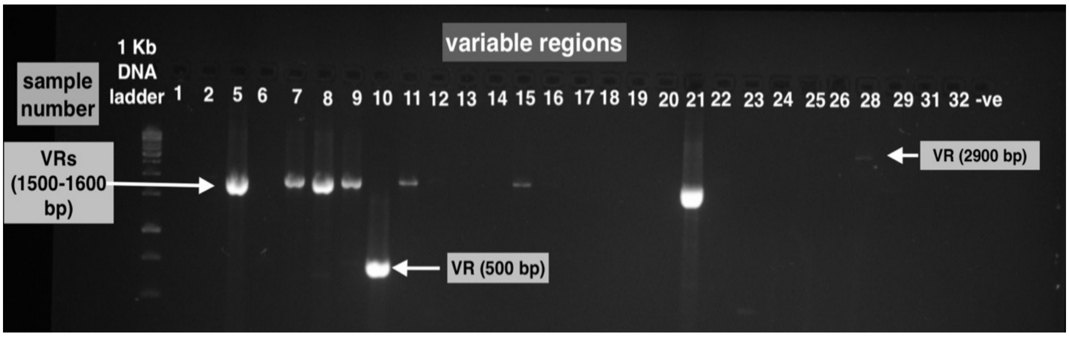

(B)

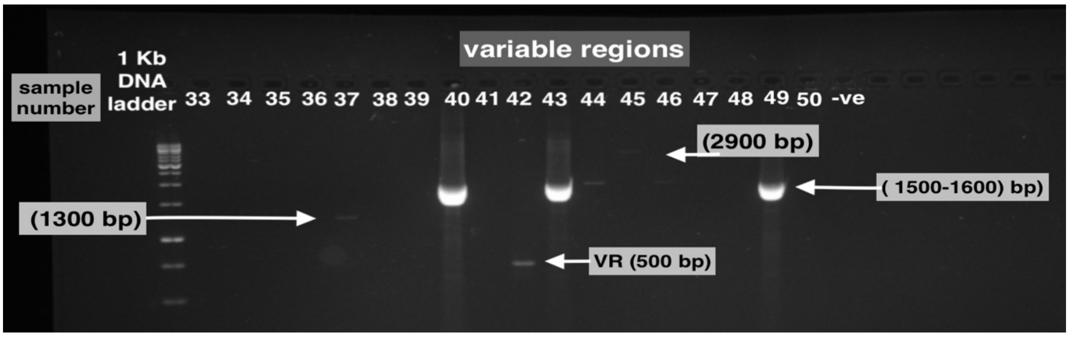

Supplementary Figure S2. Agarose gel electrophoresis of variable regions genes in 50 *K. pneumoniae* isolates.

(A) and **(B)**, lane (1) 1 Kb DNA ladder, lane (2-29) *K. pneumoniae* isolates in **A**, and (2-19) in **B**. (-ve) negative control for the variable gene.

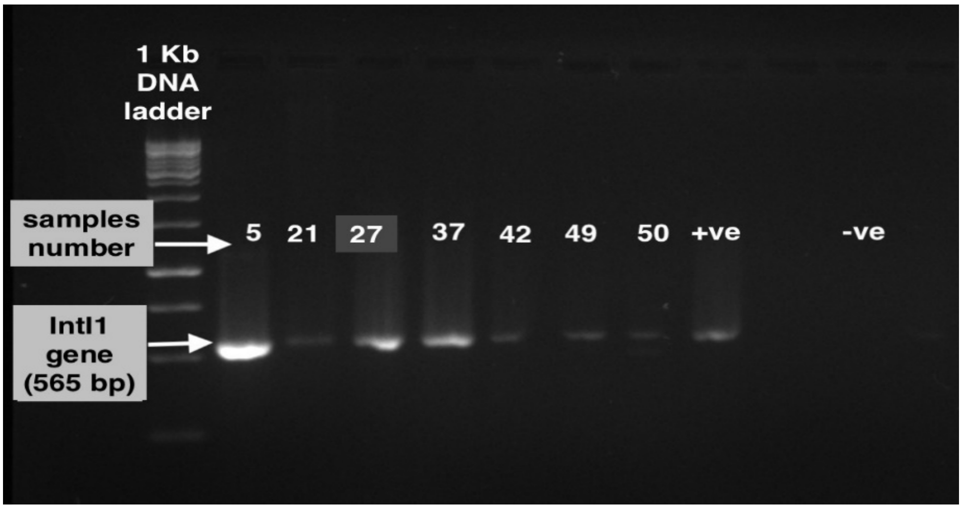

Supplementary Figure S3. Agarose gel electrophoresis of class 1 integron gene in trans-conjugant colonies from the conjugation experiment (n=7). lane (1) 1 Kb DNA ladder, lane (2-8) *intI1* as conjugation experiment products (+ve) positive control, and (-ve) negative control.
